# Integrative Multi-Omics Analysis Reveals Host Regulatory and Immune Networks with Inferred Dysbiosis Relevance in Colorectal Cancer

**DOI:** 10.3390/jcm15187131

**Published:** 2026-09-14

**Authors:** Huda Altoukhi, Nawal H Siddig, Nawal Al-Hoshani, Mohammed Y. Refai, Fahd MF Aldowsari, Tariq Aziz

**Affiliations:** 1 Radiotherapy Unit, Radiology Department, King Abdulaziz University Hospital, Jeddah 21589, Saudi Arabia; 2 Department of Mathematical Sciences, College of Science, Princess Nourah bint Abdulrahman University, P.O. Box 84428, Riyadh 11671, Saudi Arabia; 3 Department of Biology, College of Science, Princess Nourah bint Abdulrahman University, P.O. Box 84428, Riyadh 11671, Saudi Arabia; 4 Department of Biological Sciences, College of Science, University of Jeddah, Jeddah 21493, Saudi Arabia; 5 Molecular Microbiology and Infectious Diseases Research Unit, University of Tabuk, Tabuk 71491, Saudi Arabia

**Keywords:** colorectal cancer, microbial dysbiosis, host microbiome interactions, immune remodeling, tumor microenvironment, gut microbiome, multi-omics integration

## Abstract

**Background:** Colorectal cancer (CRC) remains one of the leading causes of cancer-related mortality worldwide. Increasing evidence suggests that intestinal microbial dysbiosis contributes to colorectal tumorigenesis by reshaping host molecular signaling and the tumor immune microenvironment. However, the molecular mechanisms linking microbiome-associated alterations to host regulatory networks and disease progression remain incompletely understood. **Objective:** This study aimed to identify the microbiome-associated molecular regulators and immune modulators involved in colorectal cancer through an integrative multi-omics systems biology approach. **Methods:** Publicly available transcriptomic datasets were analyzed to identify differentially expressed genes, followed by functional enrichment, protein-protein interaction network construction, hub gene prioritization, immune infiltration profiling, survival analysis, and multi-omics characterization. **Results:** The identified hub genes represent hypothesis-generating host candidates for further mechanistic and clinical validation. These genes occupied key positions within host regulatory networks and were significantly associated with adverse clinical outcomes and altered CD8^+^ T-cell infiltration, suggesting their involvement in immune remodeling within the tumor microenvironment. Multi-omics characterization demonstrated that PTEN and SMAD4 alterations were predominantly associated with genomic deletions, whereas SMAD2 dysregulation was associated with transcriptomic and gene-dosage variation. Although the overall mutational burden of the identified hub genes was not significantly associated with disease-free survival (*p* = 0.878), these molecular regulators showed associations with immune-cell infiltration and CRC-related pathways. **Conclusions:** Collectively, our findings provide a system-level framework describing the associations between dysbiosis-relevant host pathways, CRC-related regulatory networks, and immune responses.

## 1. Introduction

Despite the fact that colorectal cancer (CRC) is a huge burden in global morbidity, it still reflects a multifaceted interplay of genetic changes, chronic colonic inflammation, immune system dysfunction and environmental factors. The role of gut microbial dysbiosis in CRC pathogenesis has been suggested to involve disruption of the barrier function, changes in microbial metabolism, genotoxic activity, oxidative stress and chronic pro-inflammatory signaling [1,2]. These processes can alter the expression of host genes and alter the tumor immune microenvironment, which in turn can affect tumor-cell proliferation, immune evasion, disease progression, and responsiveness to therapy [3].

Colorectal cancer (CRC) is a significant public health problem that is influenced by multiple genetic changes, chronic intestinal inflammation, immune dysfunction and environmental triggers. Recent findings point to the role of gut microbial dysbiosis in CRC pathogenesis via impairment of gut barrier integrity, dysregulation of microbial metabolism, genotoxicity, oxidative stress, and persistent pro-inflammatory signaling [4]. There are multiple mechanisms by which microbes contribute to CRC pathogenesis. DNA damaging bacterial toxins could cause DNA damage, and changes in the synthesis of short-chain fatty acids, secondary bile acids and other bacterial metabolites could influence epithelial metabolism, regulation of chromatin structure, and inflammatory pathways. Disruption of intestinal barrier integrity could lead to greater exposure to microbial products and the activation of host pattern recognition pathways. The mechanisms indicate that alterations in the host transcriptome related to oxidative stress, DNA-damage responses, cytokine signaling and cell-cycle regulation may be indicators of microbiome disruption [5,6].

The tumor immune microenvironment is a crucial mediator of the link between microbial dysbiosis and CRC. Dysbiosis-related inflammatory and metabolic signals may affect the modulation of cytokine production, the recruitment of immune cells, and the antitumor effect, including that of CD8^+^ T cells [7]. The role of the host regulatory genes that link dysbiosis-responsive pathways to immune remodeling and CRC progression, however, are not completely understood. Previous studies have typically investigated the composition of microbes, individual genetic alterations, or immune biomarkers separately, without taking all of these into account within a single molecular network of the host organism [8,9]. Despite these observations, the link between microbial disruption, host-gene regulation and CRC-associated immune remodeling is not well characterized. Most studies that are available focus on either microbial composition or host molecular changes, with a relatively small number combining host transcriptomic, network, genomic, clinical, and immune-infiltration data. Thus, the host regulatory signaling nodes that are potential links between dysbiosis responsive processes and CRC progression are still unknown. The identification of such nodes could lead to novel experimental hypotheses about the mechanism of CRC associated with the host [10].

The current study aimed to discover host genes that are located at the nexus of CRC-associated transcriptomic changes and host pathways likely to be affected by microbial dysbiosis. These prioritized genes were then analyzed for network centrality, expression patterns, association with prognosis, genomic alterations and association with CD8^+^ T-cell infiltration. This integrated analysis aimed at revealing their potential relevance as host candidates within dysbiosis-associated CRC mechanisms [11,12].

Identification of central host regulators may help elucidate the connection between inflammatory, metabolic, oxidative stress, DNA damage and immune processes which are possibly linked to dysbiosis and the molecular changes seen in CRC. These regulators could serve as mechanism-based connections between the changes in the intestinal environment and host transcriptional or immune responses, as well as identify priorities for further experimental examination. However the present study does not provide any information on the abundance of microbes, 16S rRNA, metagenomics, metabolomics or microbiome-derived molecular information. In this context, the identified genes should be viewed as hypothesis-generating host candidate genes that are suggested to be relevant to dysbiosis-responsive pathways, not as experimentally confirmed microbiome-dependent biomarkers or therapeutic targets. Further research is needed to establish direct host–microbiome relationships and elucidate their biological and clinical relevance, and to incorporate paired tumor transcriptomics with microbial profiling, functional experiments, and independent cohorts of patients [13].

## 2. Materials and Methods

### 2.1. Dataset Acquisition

The gene-expression data for the microarray was downloaded from Gene Expression Omnibus (GEO) (https://www.ncbi.nlm.nih.gov/gds, accessed on 2 March 2026) under accession number GSE110224. It consists of 34 samples from 17 patients: 17 primary colorectal adenocarcinomas and 17 patient-matched normal colon tissues. The GPL570 Affymetrix Human Genome U133 Plus 2.0 Array was used for expression profiling. The processed expression matrix and sample annotations were downloaded for further analysis. Boxplots and density plots were used to ensure normalization of expression distributions and to detect possible technical inconsistencies [14].

### 2.2. Identification of DEGs

All paired colorectal tumor and normal samples were analyzed using GEO2R (https://www.ncbi.nlm.nih.gov/geo/geo2r, accessed on 2 March 2026) to compare the expression matrix generated by processed microarray. The results obtained from the probes were mapped to the corresponding gene symbols and differentially expressed genes were identified by applying the thresholds of adjusted *p*-value < 0.05 and log_2_ fold change > 1.0. The filtering protocol was strict, favoring those transcripts showing an adjusted *p*-value < 0.05 and |log_2_ fold change| > 1.0. Genes with log_2_ fold change > 1.0 were classified as upregulated, whereas genes with log_2_ fold change < −1.0 were classified as downregulated. To validate the integrity of the processed dataset, various diagnostic plots, such as expression distribution and mean variance trends, were created and evaluated for each set of samples. This method enabled the determination of a total of 7485 DEGs [15,16].

### 2.3. Identification of Host Genes with Potential Dysbiosis-Relevant Functions

The list of high-confidence genes was analyzed using the DAVID Bioinformatics Resources (version 2021) (https://davidbioinformatics.nih.gov/tools.jsp, accessed on 4 March 2026) which were annotated, categorized, and selected on the basis of their relevance in relation to the gut microbiome. The functional enrichment analysis was carried out within GO categories such as biological process, cellular component, and molecular function. A total of 7485 DEGs were obtained. Those terms with Benjamini–Hochberg-adjusted *p*-value < 0.05 and fold enrichment > 1.5 were kept. The annotations comprising the virome interaction, interferon response, oxidative stress, and DNA-damage response were used to determine dysbiosis relevance. A total of eight annotations were found, yielding 593 gene–term associations (Supplementary Table S1). These genes were believed to represent genes of the host response process with relevance to microbial dysbiosis instead of genes derived from the microbes themselves. Microbiome relevance was inferred through functional annotations related to host–microbial interaction, immune signaling, inflammation, oxidative stress, and dysbiosis-responsive processes. No microbial abundance, 16S, metagenomic, or microbiome-derived molecular data were analyzed. Therefore, this screening identifies host candidates with inferred dysbiosis relevance rather than directly demonstrated host–microbiome associations [17].

### 2.4. Functional Enrichment Analysis

The gene set enrichment analysis was conducted via the Metascape tool (https://metascape.org, accessed on 6 March 2026) to highlight biological themes in several biological ontologies such as the GO Biological Process, KEGG Pathway, and Reactome pathway. Differentially expressed genes were selected according to their enrichment factor > 1.5, overlap ≥ 3 genes, and adjusted *p*-value < 0.01. To avoid redundancy in the resulting biological signatures, biological terms with related meanings were clustered together based on a Kappa-test score cut-off of 0.3. This enabled the conversion of a wide array of transcriptomic data into a well-defined network of biological pathways [18].

### 2.5. Protein–Protein Network Construction

In order to investigate the interactions among these proteins, both physical and functional, a PPI network was developed using the STRING database (version 12.0) (https://string-db.org, accessed on 6 March 2026). The search was limited to homo sapiens species, and all interactions with a combined score of greater than 0.700 were considered to reduce the number of false-positive interactions in the network. Such a strict threshold for the interaction network was developed based on information derived experimentally, from curated databases, and from co-expression [19,20].

### 2.6. Identification of the HUB Genes

To identify the key genomic players in such an intricate interactome, the CytoHubba plug-in (https://apps.cytoscape.org/apps/cytohubba, accessed on 8 March 2026) in the Cytoscape software program (version 3.10) (https://cytoscape.org) was employed. Degree, MNC, and Bottleneck centrality were calculated independently for each node. The final candidates were selected based on the complementary measures, but ranked based on the concordant high ranking across these measures, which reduces dependence on a single network metric. Ten of the highest genes for consensus were kept as a practical subset for downstream analysis, but not as a significant cut-off. Hence, these genes are called prioritized network hub genes, and the less prioritized genes are not considered biologically irrelevant. The identified genes were selected through differential-expression filtering and concordant PPI-network ranking rather than prior classification as microbiome-related genes. Therefore, they represent central host regulators within the dysbiosis-associated network, not canonical microbiome genes [21,22].

### 2.7. Survival Analysis

The role of these candidate hub genes in predicting the prognosis of patients was determined using GEPIA 3 (https://gepia3.bioinfoliu.com, accessed on 12 March 2026), a web server based on standardized TCGA-COAD datasets. In order to determine the prognostic value of candidate hub genes, overall survival (OS) and disease-free survival (DFS) were evaluated for the patients based on high versus low gene expression levels. The cut-off value used to segregate the patient groups was the median expression value. The statistical significance of the results was established by the log-rank test, whereas Hazard Ratio (HR) values along with their 95% confidence intervals were determined using the Cox Proportional Hazards model. Through these analyses, it became possible to identify specific hub genes, including TGFBR2 and SMAD2, which could serve as valuable markers of disease prognosis and risk factors in colorectal cancer patients [23].

### 2.8. Clinicopathological and Cross-Platform Expression Validation

The clinical–pathological and platform-independent significance of the selected hub genes was validated through the GEPIA 3 (https://gepia3.bioinfoliu.com, accessed on 12 March 2026) web-based software through the analysis of transcriptomic profiles in TCGA-COAD/READ patient databases and the GTEx datasets. The differential expression of these selected gene hubs was assessed between CRC tumors and normal samples on the basis of the standardized log_2_(TPM + 1) method. Also, the relationship between the level of expression of these genes and their correlation with disease progression at different pathological stages (Stages I to IV) was considered. Significance was assessed using one-way ANOVA (*p* < 0.05) [23].

### 2.9. Statistical Validation of mRNA Overexpression

The mRNA overexpression analysis was conducted statistically using the GEPIA 3 website (https://gepia3.bioinfoliu.com, accessed on 14 March 2026), where TCGA-COAD datasets were combined with the baseline values for the GTEx normal mucosa dataset. The differences in expression levels between the normal and cancerous tissues were estimated using the one-way ANOVA test. Because this analysis specifically examined mRNA overexpression, a directional log_2_ fold change > 1.0 threshold was applied. The expression values from all platforms were adjusted using a log_2_TPM + 1 normalization strategy. Thus, the hub genes, including SMAD2 and TP53, were found to be significantly and consistently overexpressed in colorectal cancer compared to normal tissues [23].

### 2.10. Immune Infiltration Analysis

In order to analyze the interaction between the expressions of the hub genes and the tumor immune microenvironment, an analysis of immune infiltration in the three COAD groups was performed using the TIMER 2.0 (Tumor Immune Estimation Resource) online server (https://compbio.cn/timer2, accessed on 16 March 2026). The amount of CD8+ T cells was estimated in TCGA-COAD using various methods of deconvolution, such as TIMER, CIBERSORT, and MCPCOUNTER. Partial correlation analysis with Spearman’s correlation coefficient was used to estimate the connection between the expression of the selected hub genes and the infiltration of immune cells with respect to the percentage of tumor purity. Statistical significance was established if *p* < 0.05 [24].

### 2.11. Somatic Mutation and Copy Number Alteration Analysis

Analysis of somatic mutations and copy number variations in the hub genes was conducted via the cBioPortal for Cancer Genomics website (https://www.cbioportal.orgm accessed on 17 March 2026). The TCGA PanCancer Atlas COAD cohort was selected. Various types of genomic changes, such as missense mutation, truncation mutation, amplification, and deep deletion, were analyzed. The oncoprint graph was drawn based on the alterations found in hub genes within CRC [25].

### 2.12. Genomic Alteration and CNV Expression Correlation Analysis (GSCA)

To measure the correlation between the CNV expression profile and the gene expression level of the selected hub genes, the GSCA database was utilized (http://bioinfo.life.hust.edu.cn/GSCA/, accessed on 18 March 2026). Both CNV amplification and deletion ratios, as well as the Pearson correlation between CNVs and gene expression levels, were analyzed, with a statistical significance criterion of *p*-value less than 0.05 [26].

### 2.13. Survival Analysis Based on Genomic Alterations

Kaplan–Meier survival analysis was performed in cBioPortal (https://www.cbioportal.org, accessed on 20 March 2026) to assess the potential importance of genome-wide changes in hub genes for predicting prognosis. Patients were categorized into those having hub gene mutations and those not having hub gene mutations, and their overall survival rates were analyzed using the log-rank test, with statistical significance at the *p* < 0.05 level [25].

## 3. Results

### 3.1. Transcriptomic Data Landscape and Sample Selection

The transcriptome profile landscape was analyzed using the GSE110224 dataset, which includes 17 paired samples from patients with colorectal cancer. The initial analysis of the data showed a clear molecular distinction between the diseased state and the normal state through the use of PCA, indicating significant differences across the principal component axes. Thus, it is assured that the genes selected will be reliable and form a good basis for further analysis.

### 3.2. Exploration of Differentially Expressed Genes (DEGs)

**Figure 1:**
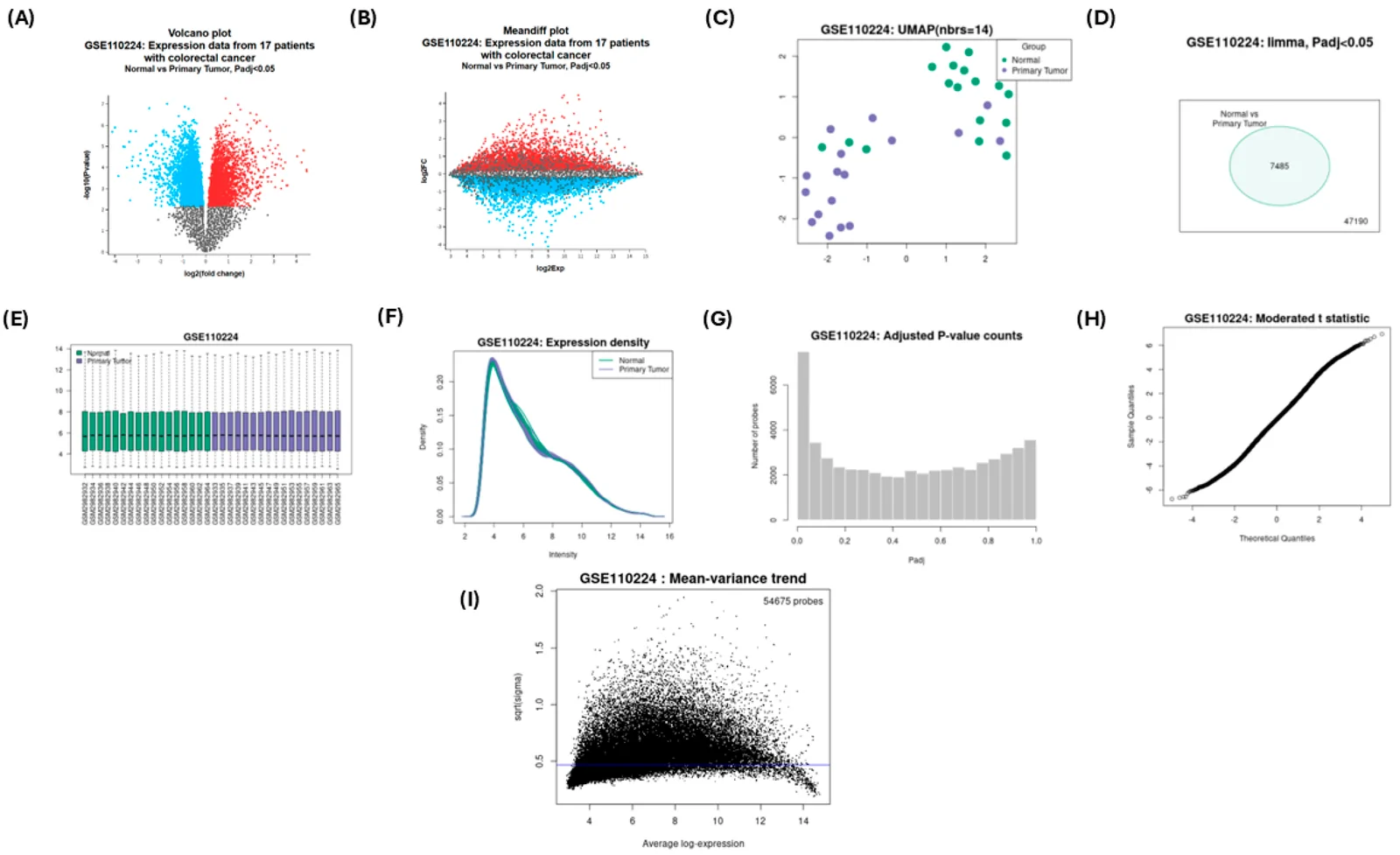
Differential expression analysis and quality validation of the GSE110224 dataset. (**A**,**B**) Volcano and MA plots showing significant transcriptional shifts (*p*-adj < 0.05). (**C**) UMAP plot demonstrating clear separation between tumor and normal groups. (**D**) Venn diagram identifying 7485 DEGs. (**E**,**F**) Boxplot and density plots confirming normalized expression across samples. (**G**–**I**) Statistical diagnostics including *p*-value distribution, Q-Q plot of moderated t-statistics, and the mean-variance trend.

Transcriptome profiling of colorectal cancer was systematically conducted using 17 pairs of tumor tissues and matched normal mucosa from the GSE110224 collection. The volcano and MA plots displayed a significant difference in transcription between CRC and matched normal tissues (Figure 1A,B). The tumor and normal samples were separated by UMAP analysis (Figure 1C) and the identified DEGs were summarized by a Venn diagram (Figure 1D). Normalization revealed similar expression distribution in boxplot and density analysis (Figure 1E,F). The *p* value distribution and the Q–Q plot and mean–variance trend was also used for statistical and quality-control analysis of the data (Figure 1G–I).

### 3.3. Identification of Microbiome-Related Gene Signatures

**Figure 2:**
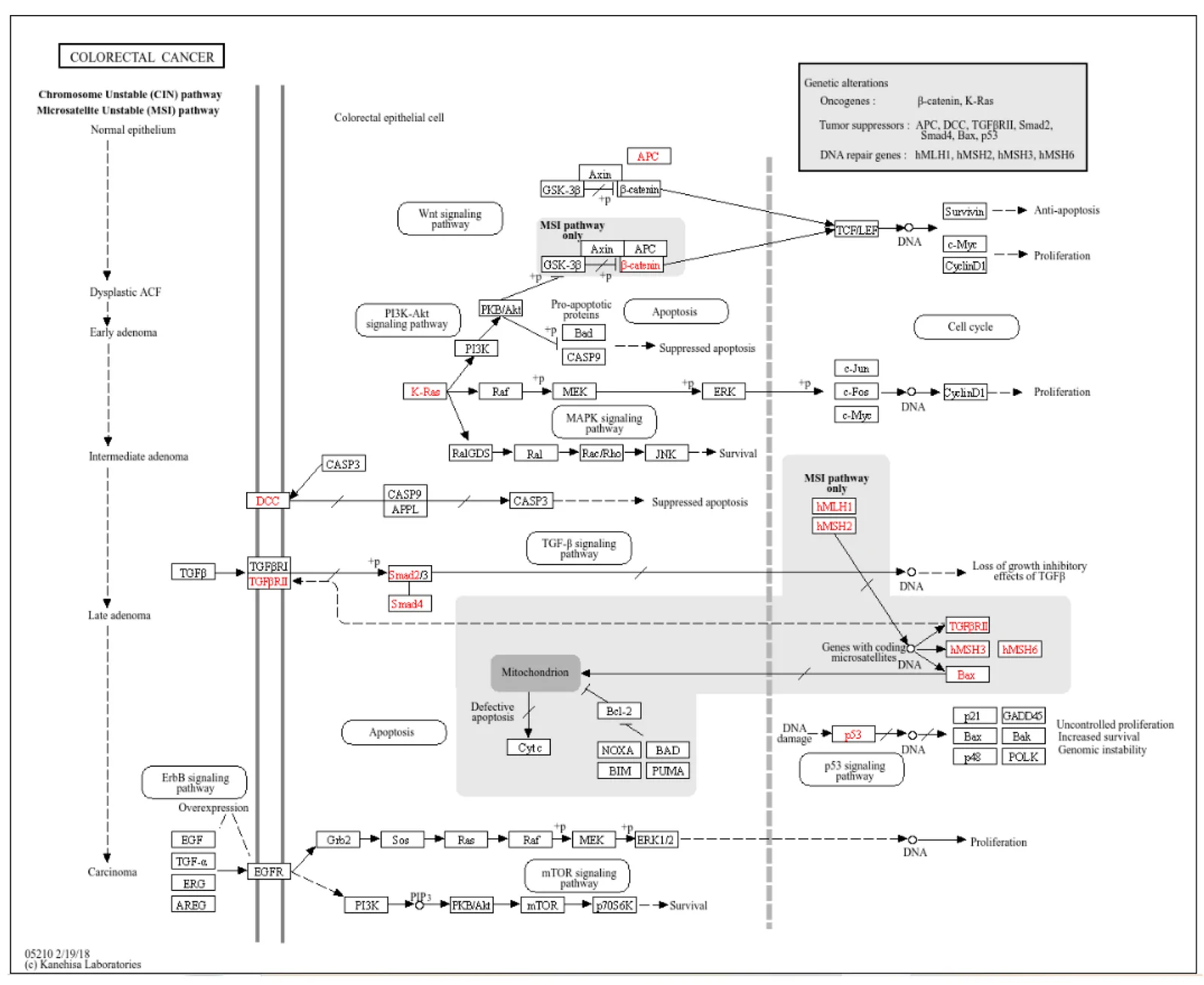
Integrated mapping of transcriptomic signatures onto colorectal cancer pathogenesis pathways. The schematic illustrates the progression from normal epithelium to carcinoma via the CIN and MSI pathways. Highlighted are key regulatory nodes—including the Wnt, PI3K-Akt, MAPK, and TGF-beta signaling networks—where differentially expressed genes (DEGs) interface with host cellular processes. Red-labeled components signify critical genetic alterations and host regulatory points associated with proliferation, genomic instability, and apoptosis-related pathways. Their direct regulation by microbial factors was not evaluated.

In order to identify the molecular interaction, point between host gene expression and the effects of microbes, the 7485 differentially expressed genes (DEGs) obtained through the analysis were overlapped with known signaling pathways in colorectal cancer, particularly concentrating on the CIN and MSI pathways. The integration of transcriptome profiling with the existing pathway structure indicates remarkable disturbances in key pathways that are under the control of oncogenic genes, such as K-Ras and beta-catenin, and tumor-suppressor genes, such as APC and p53. Eight statistically significant annotations were identified by DAVID that might be relevant to microbial dysbiosis: host–virus interaction, virus–receptor activity, type I interferon response, type II interferon response, oxidative-stress response, and DNA-damage response. These annotations included 593 gene–term associations (Supplementary Table S1). Ten genes were identified as hub genes, with TP53 connecting to host–virus interaction, oxidative-stress response, and DNA-damage response, and MSH6 connected to host–virus interaction. All the other hub genes were selected by differential expression and PPI-network centrality instead of direct connection to the microbiome. Hence, these results corroborate functional relevance to dysbiosis-related host responses, but do not provide direct evidence of microbial regulation. Host-response gene sets of potential relevance to dysbiosis-associated immune, oxidative-stress and DNA-damage processes were identified through retained annotations. They do not prove that the genes are directly controlled by microbes, or that they are responsible for genomic instability or for immune escape (Figure 2).

### 3.4. Functional and Pathway Enrichment of Candidate Genes

Functional analysis of the identified DEGs was carried out with the help of Metascape software v3.5.20260701 using different ontologies such as Reactome, GO Biological Processes, and WikiPathways. The pathway enrichment analysis data are provided in Table 1. According to the obtained data, cell cycle disruption and immune responses can be identified as the major functional categories. The highest level of enrichment was achieved for cell cycle (R-HSA-1640170) and mitotic cell cycle (GO:0000278) pathways, containing 217 and 149 DEGs, respectively. It is noteworthy that the identified significant pathway shift is related to the loss of cell cycle regulation due to the enrichment of RB in cancer (WP2446) and M phase (R-HSA-68886) pathways. In addition to proliferation-related processes, significant enrichment was noted for pathways involved in host–microbiome interactions, including cytokine signaling in the immune System (R-HSA-1280215) and neutrophil degranulation (R-HSA-6798695). Such data indicate that transcriptomic changes may be associated with the influence of the inflammatory microenvironment and microbiome on the development of CRCs. In addition, a significant number of genes were associated with cellular response to stress (R-HSA-2262752) and RNA metabolism (R-HSA-8953854) pathways. Table 1 below represents the top enriched biological pathways and processes identified by Metascape.

In order to determine the structural organization among the DEGs, a protein–protein interaction (PPI) network was built and further separated into functional modules. Modules are defined as clusters of highly connected genes, representing the gene groups that most probably work together in some particular niche. As stated in Table 2, the results show a wide range of cellular phenotypes, many of which belong to stem cell phenotypes and specific tissue niches. It is important to note that the network shows the presence of functional modules belonging to duodenal differentiating stem cells (M40025) and gastric isthmus cells (M40010). Thus, the enrichment with these gastrointestinal-related cellular phenotypes may reflect the fact that CRC transcriptional reprogramming includes the upregulation of genes that participate in mucosal regeneration and differentiation processes. Moreover, the presence of mesenchymal-related modules such as pancreas mesenchymal stromal cells (M39175) and ovary follicle stromal cells (M41703) can show that there is considerable interest in microenvironment remodeling.

**Figure 3:**
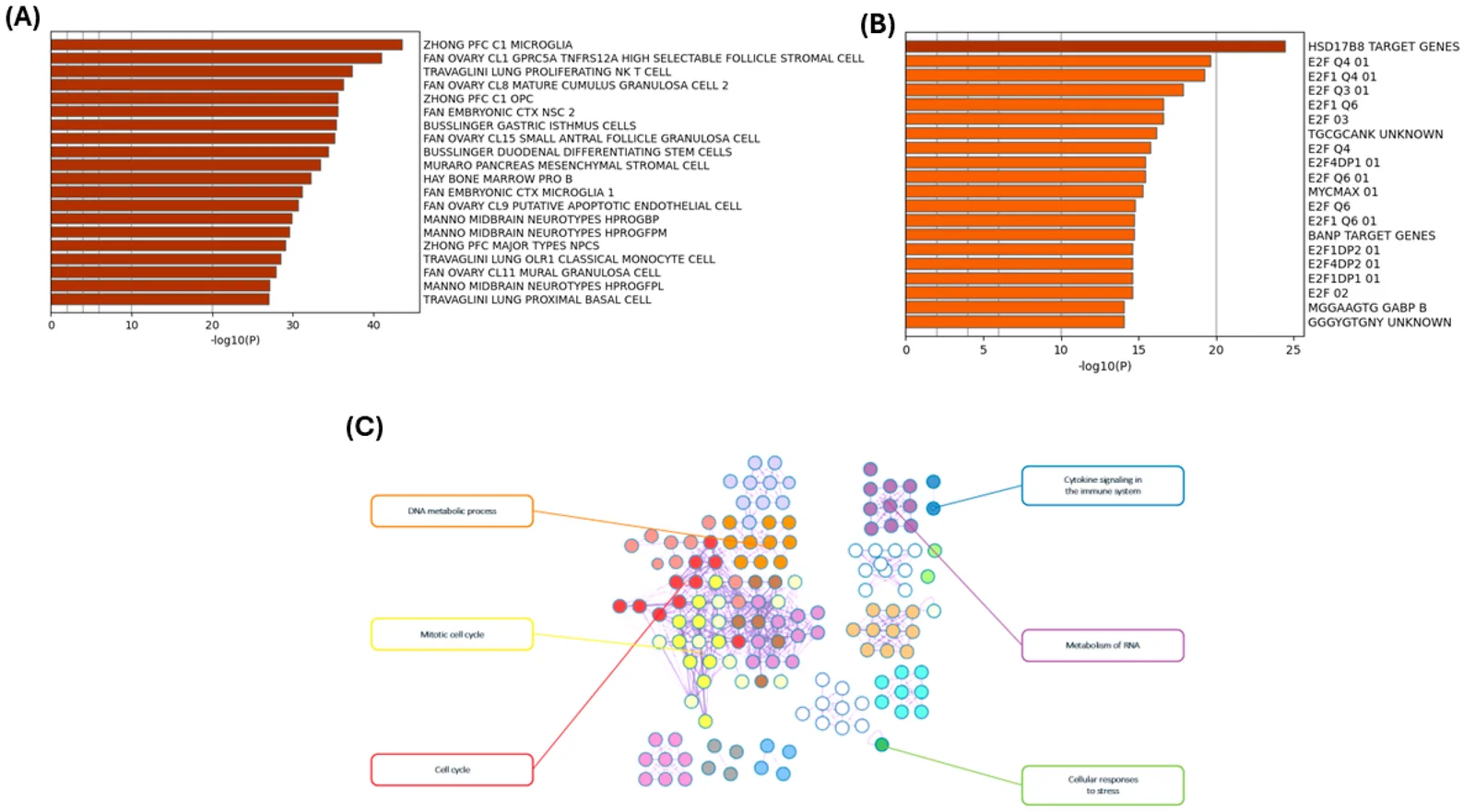
Functional enrichment and network analysis of candidate genes. (**A**) Bar plot of the significantly enriched biological pathways, ranked by statistical significance. (**B**) Enrichment of transcription-factor-target annotations associated with the candidate genes. (**C**) Network of enriched biological terms, with node colors indicating functional clusters and edges representing term similarity based on shared genes.

The results obtained from the enrichment study were presented in a bar chart showing the enriched terms according to their *p*-values, as shown in Figure 3A. Analysis of regulatory motifs was carried out to determine the transcription factors (TFs) that could be responsible for the changes in the expression pattern. As highlighted in Table 3, a significant prevalence of E2F (E2F1, and E2F4) TFs was found. The E2Fs were detected consistently across several regulatory modules (such as M102, M8998, and M10526). The E2F family showed an extremely high level of statistical significance with the Log_10_ *p* = −20.00 value, which is consistent with previous observations on the disruption of the cell cycle and suggests that there is a concerted effort from this family of transcription factors to promote mitotic progression. Moreover, the identification of target genes for MYCMAX (M12874) and BANP (M29900) highlights the complexity of this network, where these two TFs are supposed to play a role as master regulators of cancer cell metabolism and genome stability. It can be assumed that the candidate genes are subjected to strict control by a selected set of oncogenic TFs.

The functional connection between various biological pathways was studied using the network representation approach for the enrichment of different terms. As shown in Figure 3B, the nodes correspond to the significantly enriched biological pathways, and the edges depict the genetic similarity among them. The resultant structure indicates the presence of a very well-connected core cluster, wherein the pathways corresponding to cell cycle (mitotic cell cycle, M phase, and DNA metabolism) are densely interconnected. This signifies the presence of an orchestrated mechanism involving the process of genome replication and division that is observed in the case of malignancy. This core cluster is surrounded by separate clusters corresponding to specific biological pathways, such as the cytokine signaling pathway, the immune system, and the metabolism. While each of these networks is separated from the others in terms of functionality, they are still connected via a small number of multifunctional genes. Figure 3C shows the functional organization of the significantly enriched terms. The main clusters encompass cell-cycle/DNA-repair, immune–cytokine/interferon, oxidative-stress, and metabolic processes. While this network does not explicitly represent microbial taxa or gene–microbe interactions, it shows how host pathways converge and might become dysregulated during gut microbial dysbiosis. Specifically, signals from the microbiome can interact with pathways of proliferation and DNA damage, which are all relevant to colorectal tumorigenesis, as well as inflammatory, immune, and oxidative stress pathways.

### 3.5. Construction of the Interactive Protein Network

**Figure 4:**
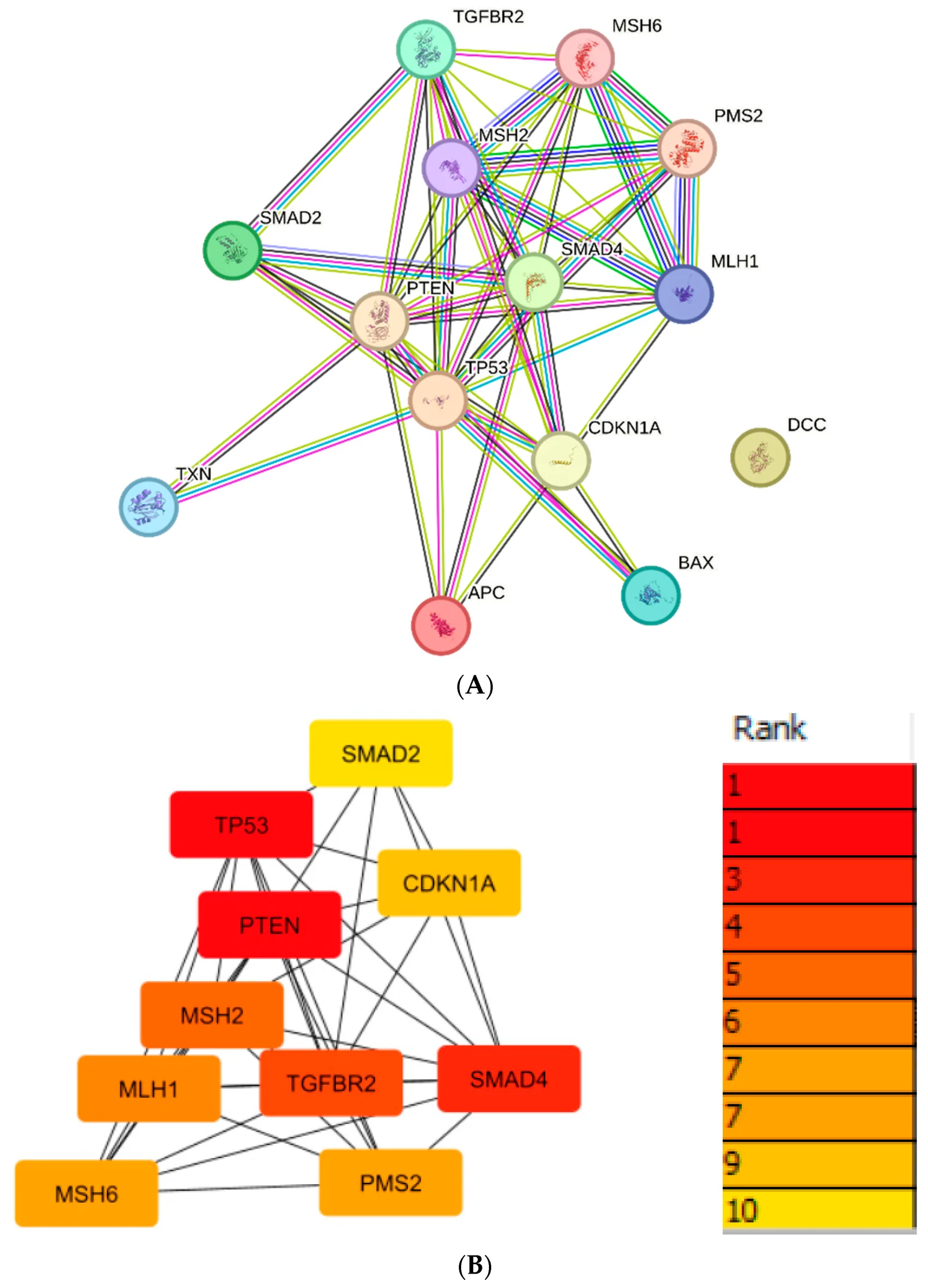
(**A**): Protein–protein interaction (PPI) network of key hub genes in colorectal cancer. The network highlights the physical and functional interactions between identified differentially expressed genes. Nodes represent individual proteins, with their structural representations depicted within the circular boundaries. Edges indicate validated interactions, where the multi-colored lines reflect the diverse evidence types (e.g., experimental, co-expression, and database-curated). The central cluster features prominent tumor-suppressors (TP53, PTEN, APC) and DNA repair enzymes (MLH1, MSH2), identifying them as critical nodes in the CRC molecular architecture. (**B**): Identification and topological analysis of hub genes. The interaction network displays the top-ranked genes prioritized by degree centrality and connectivity. The color intensity (red to yellow) represents the relative importance of each node within the network, with TP53, PTEN, and SMAD4 identified as the central regulatory hubs. Nodes and edges represent proteins and their physical/functional interactions, respectively, emphasizing the highly interconnected nature of the DNA repair and TGF-beta signaling axes.

A protein–protein interaction (PPI) network was constructed to prioritize the central network nodes within the dysregulated transcriptomic landscape of CRC. The resulting interactome, visualized in Figure 4A, reveals a highly interconnected core of hub genes and CRC-associated proteins potentially relevant to dysbiosis-responsive host pathways. Central to this network are master regulators such as TP53, PTEN, and APC, which exhibit a high degree of centrality and serve as primary integration points for multiple signaling pathways. The density of interactions involving DNA mismatch repair proteins, specifically MLH1, MSH2, MSH6, and PMS2, highlights the functional dominance of genomic stability pathways in this cohort. Furthermore, the close physical and functional proximity of TGF-β mediators (SMAD2, SMAD4, and TGFBR2) to the central hubs suggests a coordinated crosstalk between extracellular signaling and nuclear transcriptional control. The PPI network suggests the convergence of several CRC-relevant mechanisms. TP53, PTEN, and APC regulate DNA-damage responses, PI3K–AKT signaling, and Wnt/β-catenin activity, respectively, which affect cell survival, proliferation, and apoptosis. The proteins MSH2, MSH6, PMS2, and MLH1 are responsible for mismatch repair, and their loss is associated with microsatellite instability and mutations. The network is connected to the TGF-β pathway by SMAD2, SMAD4 and TGFBR2, all of which influence the growth, invasion and progression of epithelia. In sum, the interactions between these proteins can contribute to several hallmarks of CRC, including genomic instability, uncontrolled proliferation, immune changes and invasive behavior. By mapping these high-confidence interactions, the network identifies a stabilized framework of proteins that likely involves a multifaceted orchestration of chronic inflammation, genotoxicity, metabolic reprogramming, and immune evasion.

### 3.6. Selection and Prioritization of Central Hub Genes

To narrow down the priority list of critical genes involved in colorectal cancer, a more sophisticated analysis of the protein–protein interaction network was performed in order to detect “hub genes,” which play key roles because of their topology. As illustrated in Figure 4B, the PPI map has been filtered to include the nodes with the highest connectivity, which act as bottlenecks through which all the signal pathways flow. Key hubs, such as TP53, PTEN, and SMAD4 (shown in intense red), possess the highest centrality degree and, therefore, can be considered as the key driving forces behind the malignant phenotype in this particular population. Secondary hubs, consisting of DNA mismatch repair enzymes, such as MSH2 and MLH1, and a signaling receptor, TGFBR2, show an apparent association with primary nodes. The color code, from red to yellow, corresponds to the bottleneck or degree centrality scores of each gene in the whole interactome. After identifying the subnetwork of hub genes, we are able to see a particular set of genes responsible for the transformation from stress induced by microorganisms into unrestrained cell division. The identified genes were prioritized as network hubs for subsequent expression, survival, immune-infiltration, and genomic characterization. Network centrality alone does not establish biomarker performance or therapeutic targetability.

### 3.7. Exploratory Expression and Survival Analysis of Hub Genes

**Figure 5:**
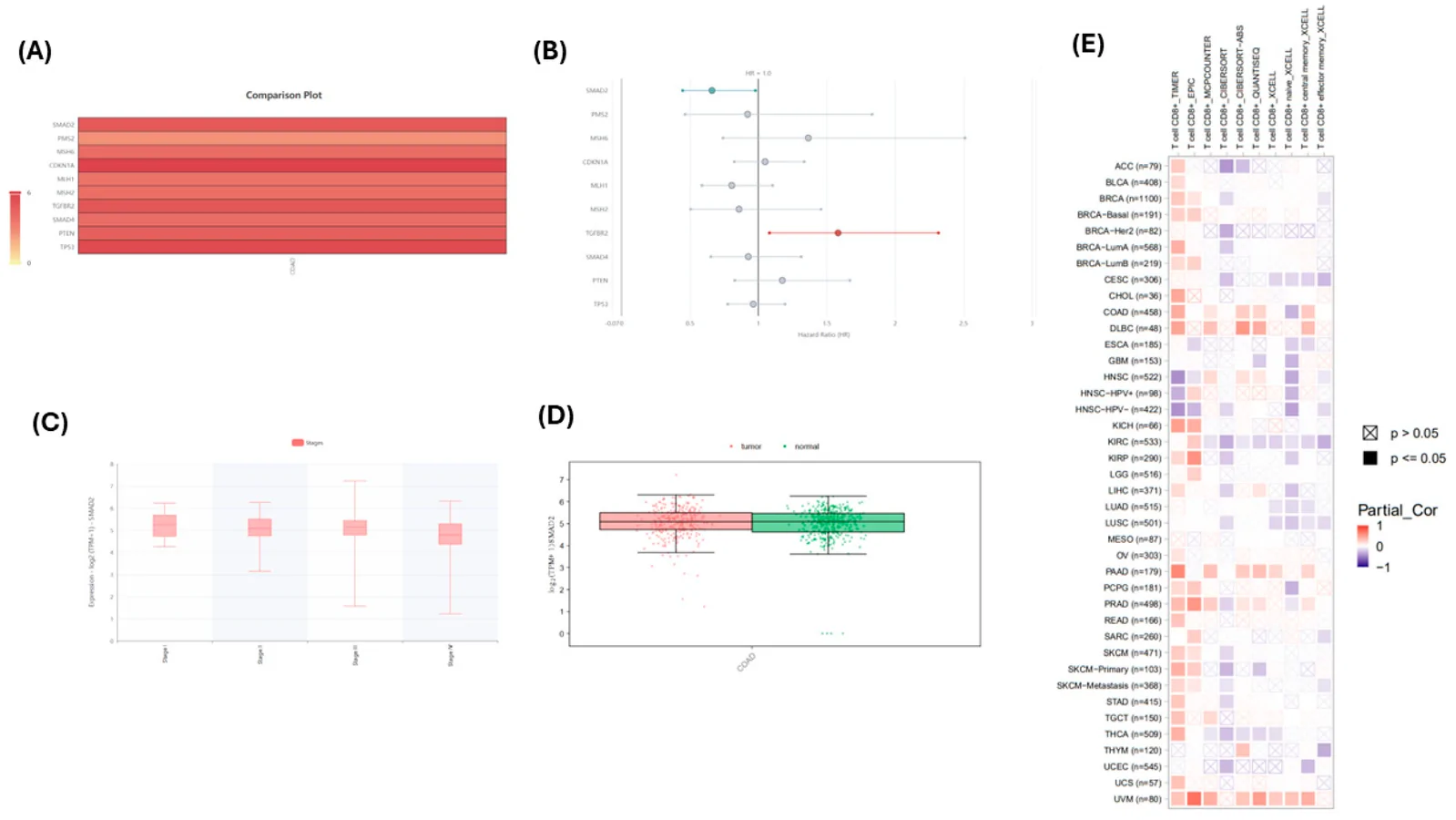
Clinical, expression, and immune characterization of the prioritized hub genes. (**A**) Expression landscape of the ten hub genes in TCGA-COAD. (**B**) Cox proportional-hazards analysis. (**C**) SMAD2 expression across CRC pathological stages. (**D**) Cross-platform comparison of SMAD2 expression between tumor and normal tissues. (**E**) Pan-cancer correlations between hub-gene expression and CD8^+^ T-cell infiltration.

Survival analysis and the assessment of differential gene expression patterns among the 10 hub genes were conducted in the COAD (Colon Adenocarcinoma) TCGA dataset. It can be observed from the comparison chart (Figure 5A) that these 10 hub genes have a consistently high level of expression. This finding supports their consideration as candidate molecular markers that require validation in independent clinical cohorts. Genes such as CDKN1A, TP53, and SMAD4, for instance, retain a relatively high intensity score, signifying a consistent contribution toward the development of colorectal cancer pathology. Their potential clinical relevance was further explored through association with overall patient survival. High expression of this core hub gene signature, especially those involved in DNA mismatch repair (e.g., MLH1, MSH2, and MSH6) and TGF-β pathway signaling (e.g., SMAD2, SMAD4), has been linked with a particular survival outcome pattern in CRC patients. Survival analyses showed heterogeneous associations across the ten hub genes. Most estimates had wide confidence intervals and limited statistical support, indicating substantial uncertainty. These results are exploratory and do not establish the genes as reliable predictors of patient outcome.

### 3.8. Cox Proportional Hazards Analysis of Hub Genes

To determine the individual effect of the hub genes on the patient survival rate, a Cox proportional hazards model was used. From Figure 5B, it is evident that the hazard ratio (HR) values of the 10 selected genes show differences in associations with mortality from colorectal cancer. The HR estimate was greater than 1.0 in case of TGFBR2, while the HR estimate was less than 1.0 in case of SMAD2. These directions are based on association, and do not necessarily represent a statistically validated or independently verified adverse or protective effect on prognosis; rather, they are exploratory directions. The implication is that the up-regulation of the gene increases the risk of poor survival rates. On the contrary, SMAD2 had a protective indication (green marker) as it had an HR value below the unit line. The other hub genes such as TP53, PTEN, and the MSH/MLH DNA repair system displayed HR values oscillating around the 1.0 line with wide confidence intervals. This suggests that, although these genes are significant drivers of carcinogenesis, their effects on the overall survival rate are influenced by other parameters.

### 3.9. Correlation of SMAD2 Expression with Colorectal Cancer Pathogenesis and Clinical Stage

To identify the gene that is most likely to exhibit a distinct protective association in Cox analysis, despite having a central position in the TGF-β-related PPI network, SMAD2 was chosen for focused exploratory analysis. SMAD2 was selected for descriptive follow-up because of its relatively stable stage-associated expression and lower genomic-alteration frequency. This analysis was exploratory and was not designed to determine the mechanism underlying its expression. This selection was not intended to imply that SMAD2 is more important than the other hub genes. The overall results of all ten hubs were analyzed using expression, survival, immune-infiltration, correlation, and genomic-alteration analysis, but validation of all candidate genes was not the scope of the present study. The expression pattern of SMAD2 (with particular emphasis on this hub gene) was studied in relation to various clinical stages to assess whether it correlated with the progress of the disease. As shown in the box plot (Figure 5C), the expression levels of SMAD2 do not change drastically between Stage I and Stage IV but remain fairly stable. SMAD2 expression showed broadly similar median levels across pathological Stages I–IV, with greater variability in some advanced-stage samples (Figure 5C). No clear stage-associated expression pattern was observed. Therefore, this descriptive analysis does not establish a stage-dependent role for SMAD2 or demonstrate its involvement in EMT.

### 3.10. Cross-Platform Validation of mRNA Expression Levels

In order to evaluate the stability of the hub genes found during the analysis, the mRNA levels of SMAD2 in tumors and normal mucosa samples in the TCGA-COAD cohort were examined. As depicted in Figure 5D, the mRNA levels of SMAD2 continue to be higher in patients with colorectal adenocarcinoma than in healthy individuals. The box plot indicates a tight expression range between the two groups, while the median level of SMAD2 in cancerous tissues is shown to be consistently superior to the baseline level of mRNA in normal mucosa. The results indicate that the same transcription profile discovered in the GSE110224 cohort was maintained in a larger dataset using a different sequencing platform. The occurrence of outliers in both groups demonstrates the natural biological variability of the TCGA cohort, but the general trend provides compelling evidence for the consistent role of SMAD2 as a reliable marker of CRC development.

### 3.11. Correlation of Hub Genes with the Immune Microenvironment

In order to determine the interaction of the identified biomarkers with the tumor immune microenvironment, a cross-cancer association study was performed using several markers of CD8^+^ T cell infiltration. As presented in Figure 5E, the hub gene signature is significantly correlated with the immune microenvironment across many cancers, such as COAD, READ, and BLCA. In both the COAD and READ cohorts, there is a robust positive partial correlation (indicated by red background) between the expression of the hub genes and the CD8^+^ T cell density using multiple methods of estimation, such as TIMER and MCPCOUNTER. The implication here is that the host-response genes with potential relevance to microbial dysbiosis and microbiome-derived genes could be interacting with the immune system to recruit more CD8^+^ T cells to infiltrate the tumors. Hub-gene expression showed significant correlations with computationally estimated CD8^+^ T-cell abundance in several cancer cohorts, including COAD and READ. The direction and magnitude of correlations varied across genes and deconvolution methods. These findings indicate expression–infiltration associations but do not demonstrate immune-cell recruitment, direct immune regulation, or treatment response.

### 3.12. Somatic Mutation Landscape of Hub Genes

**Figure 6:**
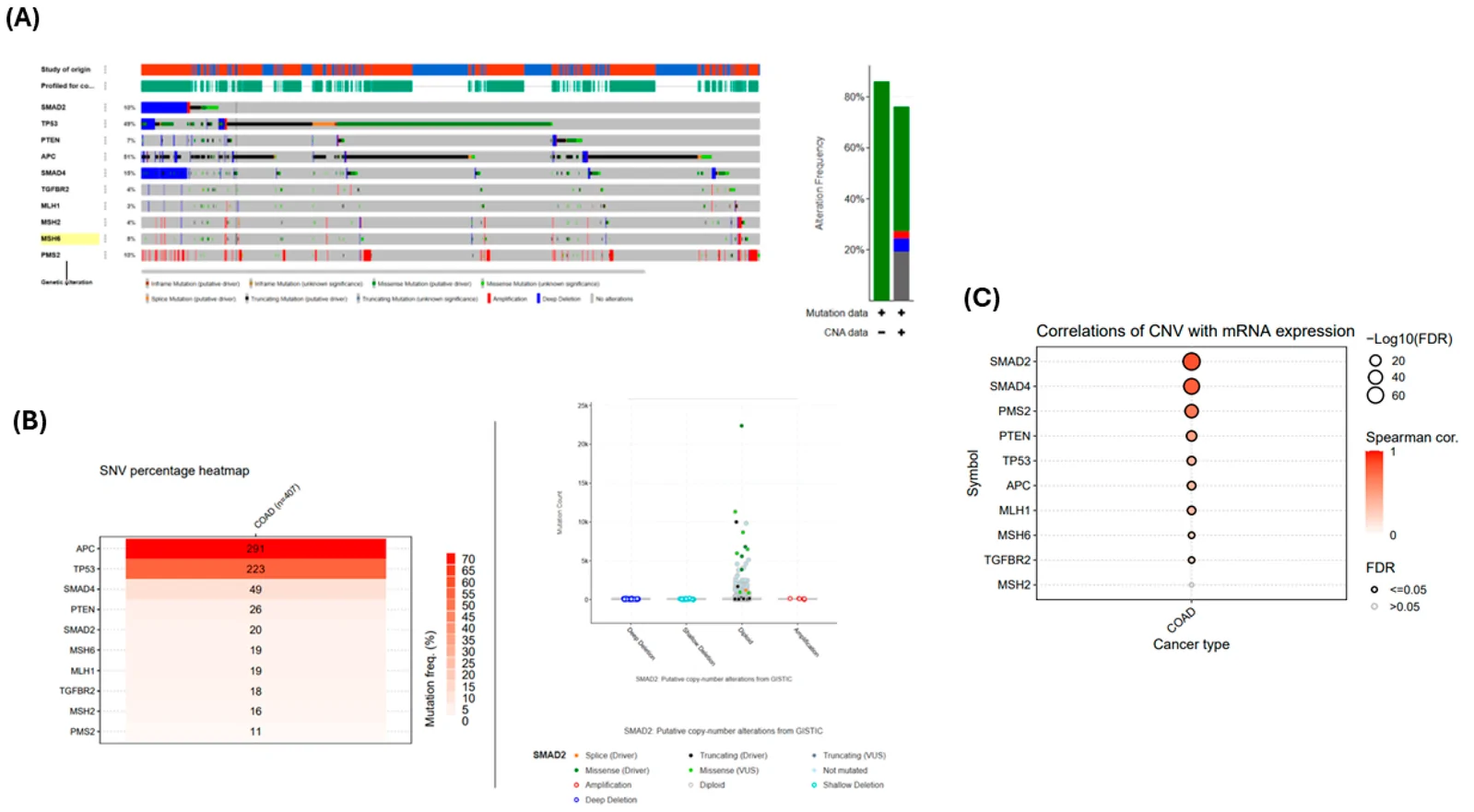
Genomic alterations and copy-number regulation of hub genes. (**A**) Somatic-mutation and CNV landscape. (**B**) Distribution of CNV types across the hub genes. (**C**) Correlations between CNV and mRNA expression in TCGA-COAD.

As indicated by the OncoPrint analysis, around 80% (1306/1634) of cases had at least one mutation in the targeted gene list, implying that there is high genomic variation amongst this group of hub genes. Amongst the hub genes, TP53 and APC were highly mutated (around 49–51%), where the majority of the alterations involved either truncation or missense mutations. PTEN and SMAD4 showed medium alteration rates of (7–15%), while the mismatch repair (MMR) genes, including MLH1, MSH2, MSH6, and PMS2, showed low but significant mutation rates (3–10%). Analysis of mutation patterns suggested that missense and truncation mutations are highly prevalent, with recurrent hotspots detected specifically within the SMAD gene family members. Co-mutation analysis also pointed towards strong co-occurrence rates between DNA repair genes and signaling hubs. These findings confirmed that the selected hub genes are recurrently mutated in colorectal cancer and are important for tumor progression both through gene disruptions and pathway-level interactions. Figure 6A below represents the genomic alteration landscape of hub genes in colorectal cancer.

OncoPrint displaying mutation and CNV frequencies across the hub gene network. While TP53 and APC dominate the mutational landscape, SMAD2 shows moderate alterations, suggesting its significant transcriptomic dysregulation is driven by microenvironmental crosstalk and microbial dysbiosis rather than primary genomic shifts.

### 3.13. Copy Number Variation and Genomic Alteration Profile of Hub Genes

The stability of the genomes of the selected hub genes was analyzed based on CNVs (Copy Number Variations). To achieve this objective, a CNV analysis was conducted using the GSCA (Gene Set Cancer Analysis) tool for the TCGA-COAD sample collection. The findings of this genomic analysis demonstrate that the genetic makeup of this set of hub genes was very different. However, it should be noted that a tendency towards heterozygous deletions predominates among the selected hub genes. In particular, it is necessary to point out that PTEN, SMAD4, and TGFBR2 are distinguished by a significant CNV burden, since they feature frequent heterozygous and homozygous deletions at a level of more than 55–60%. At the same time, genes like SMAD2 are represented by rare amplifications and deletions, which suggests that their previous detection of mRNA overexpression is controlled by another mechanism. The CNV distribution profiles are represented below in Figure 6B.

Pie plots summarize the frequency of copy number variations (CNV) for the identified hub genes. The results highlight a dominant landscape of heterozygous deletions, particularly in PTEN (60.53%), SMAD4 (63.86%), and TGFBR2 (56.32%), indicating a high degree of genomic loss in these critical tumor-suppressive pathways within the TCGA-COAD cohort.

### 3.14. Correlation Between Copy Number Variation and Gene Expression (GSCA)

In order to find the impact of genomic changes on transcriptomic expression, the correlation between CNVs and mRNA expression was studied through the GSCA interface. The data obtained showed a highly significant positive Spearman correlation among most of the hub genes within the COAD set, thereby confirming the dose-dependent regulation of their expression. Among the hub genes, SMAD2, SMAD4, and PMS2 showed higher correlations (FDR < 10^−60^; bubble size and color saturation), and it can be concluded that genomic alterations were responsible for their expression levels. Although most of the genes showed a highly significant correlation (statistical FDR0.05), only MSH2 showed no statistical correlation (FDR > 0.05). This means that other factors, like DNA methylation or stress due to microbial invasion, are involved in the expression of MSH2 in colorectal cancer patients (Figure 6C).

### 3.15. Prognostic Impact of Genomic Alterations in Hub Genes

**Figure 7:**
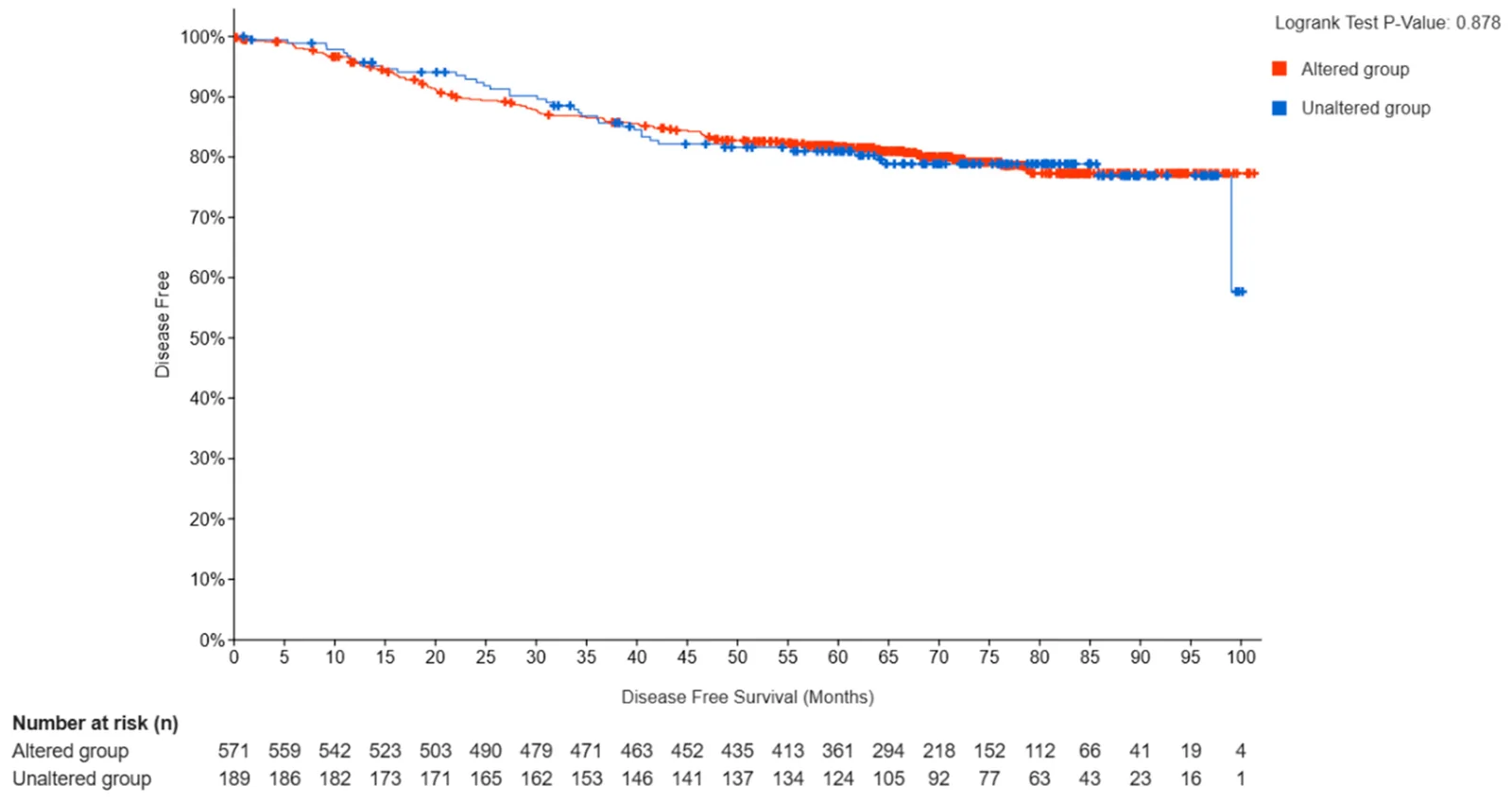
Kaplan–Meier analysis of disease-free survival based on hub gene alterations.

The Kaplan–Meier survival curves showed that there was a lot of overlap between the Altered group (i.e., patients with mutations, amplifications, or deletions in hub genes) and the “Unaltered group” over a span of 100 months. The *p*-value from the log-rank test is 0.878, which means that the collection of all these genomic alterations as a whole is not really able to predict disease-free survival rate in the TCGA-COAD dataset. This implies that while the hub genes are important drivers of tumor growth and environment shaping, their effect on patient prognosis can perhaps be better studied by analyzing their transcription level (as shown in Figure 7). No statistically significant difference in disease-free survival was detected between patients with and without genomic alterations in the ten hub genes (log-rank *p* = 0.878). Therefore, genomic-alteration status in this gene set did not demonstrate prognostic value in the analyzed cohort.

Disease-free survival according to hub-gene genomic-alteration status. Kaplan–Meier curves compare patients with at least one alteration in the ten hub genes with patients without alterations. No statistically significant difference was detected between the groups (log-rank *p* = 0.878).

## 4. Discussion

Genomic-stability and tumor-suppressor functions were suggested by the high connectivity of TP53, PTEN, and SMAD4 in CRC-related protein-interaction networks. Correlations between hub-gene expression and CD8^+^ T-cell infiltration suggest possible relationships with the tumor immune microenvironment. These associations are not, however, sufficient to conclude that the genes are responsible for the recruitment of immune cells or for transiting dysbiotic inflammation into tumor progression in the context of the microbiome [27]. The high levels of connectivity demonstrated by these core genes within the PPI network imply that they act as metabolic and signaling hubs that help in the conversion of dysbiotic inflammation to cancerous tumors [28]. Moreover, the strong link that exists between the hub gene abundance and CD8^+^ T-cell presence implies that these genetic regulators contribute to the change in tumor immune microenvironment, thereby influencing their recruitment [29]. This implies that the hub genes act as intermediary regulators within the microbiome–immunity axis, where microbiome-induced changes in gene expression determine the nature of the tumor [30].

The results of this study are consistent with the current knowledge concerning the adenoma–carcinoma sequence, although it provides a deeper understanding, taking into account the role of the gut microbiota [31]. Identified hub genes fall into oncogenic signaling pathways that are previously reported CRC and dysbiosis-related host responses [32]. The current data, however, cannot prove a causal link between microbial dysbiosis and genomic changes. Instead, what the findings suggest are possible functional connections between host gene expression, pathways involved in CRC, and biological processes involved in dysbiosis. The high incidence rate of heterozygous deletion mutations in PTEN, SMAD4, and TGFBR2 (>55%) indicates substantial genomic stress in the TCGA-COAD set, further confirming the “loss-of-function” hypothesis in colorectal cancer development. The relatively low frequency of mutation found in SMAD2 despite its high expression level hints at a non-genomic control mechanism [33,34]. TP53, PTEN, SMAD4, and SMAD2 are not canonical microbiome-associated genes, but could be convergence points in host signaling for dysbiosis. Microbial genotoxicity and inflammation may impact TP53-dependent stress responses, PTEN-regulated PI3K/AKT signaling, and SMAD2/SMAD4-mediated TGF-β signaling. These pathways are responsible for maintaining epithelial homeostasis, immune responses, and CRC progression. The present results do not support microbial regulation, but rather functional association [35,36].

These prognostic effects of TGFBR2 and SMAD2 are not contradictory to their identification as network hubs, as the two properties of the network (topological centrality and prognostic direction) describe two different biological properties. Consequently, while the canonical SMAD-dependent pathway is growth-inhibiting, the non-canonical pathway may have growth-promoting effects through the activation of TGFBR2 in advanced CRC, and this could lead to the retention of growth-inhibitory functions of the canonical TGF-β pathway in the absence of SMAD2 activity. Their impact can also vary depending on the molecular subtype of the tumor, their molecular expression, and their source of origin. The overall associations may also be influenced by cohort heterogeneity and individual-gene Cox models. These results should be considered preliminary and require confirmation in larger, stratified cohorts, and are not meant to imply that all network hubs have the same prognostic value [37].

There has also been a recent trend in computational approaches that highlight the importance of multi-omics and clinical data for precision medicine, along with microbiological data. In a recent paper, Stoia et al. explained how AI can be used to integrate these disparate data types to inform personalized antimicrobial decisions, taking into account the disruption of the microbiome by antibiotics. Their framework is relevant for gastro-renal infections, but may be instructive for future microbiome-informed oncology. The current host gene analysis will be expanded by pairing with microbial-abundance and metabolomic, treatment, and clinical data, which could lead to better patient stratification. The challenge of data heterogeneity, limited prospective validation and interpretability, however, are critical obstacles to clinical use [38].

Despite the computational robustness of this study regarding the discovery of hub genes associated with colorectal cancer, there are some limitations that need to be considered [39]. Firstly, most of the data collected during this study comes from retrospective sources such as public databases, meaning that batch effects might have been introduced by differences in the handling of samples between various studies [40]. Secondly, despite having high-confidence predictions concerning the infiltration of immune cells with TIMER 2.0 and GEPIA 3, it is worth mentioning that these results are purely in silico and do not include any kind of experimental validation (i.e., immunohistochemistry or flow cytometry). Furthermore, a link between the presence of microbial signatures and immune cell recruitment could be established, although a causal relationship could not be established without wet-lab experimentation. Due to the lack of paired microbial and host molecular data, the direct assignment of identified genes to microbial dysbiosis is limited. They are predicted to be relevant to the microbiome based on functional annotation and biological inference, but need to be confirmed with paired metagenomic, microbial-abundance, metabolomic, and host-transcriptomic data. Furthermore, GSE110224 is composed of patient-matched tumor and normal samples, but the DEG analysis was not explicitly designed to take into account patient matching. This could have influenced the estimation of variance and the subsequent DEG list, and thus is a constraint on the present analysis [41,42].

In addition to the clinical applications in prognosis, the identification of such hub genes also holds significance within the field of precision oncology. The validation of these biomarkers as key regulators of the tumor immune microenvironment would enable clinicians to rely on transcriptomic analysis in the future to identify the prognostic level of the patient [43,44]. No bacterial annotations, lipopolysaccharide, or Toll-like receptor annotations met the prespecified DAVID thresholds. In addition, there is no matched abundance data for microbes in GSE110224. Therefore, identified genes must be considered as host-response candidates specific to dysbiosis—not as microbial biomarkers—and causal gene–microbe relationships need to be substantiated with paired microbiome and host-transcriptomic data.

Further research must validate these key genes experimentally using CRISPR-Cas9 gene silencing techniques in an in vitro environment and microbiota transplantation in an in vivo model to establish the causal link between them and immune regulation. Longitudinal clinical trials must examine these gene expression profiles as potential biomarkers in liquid biopsy samples to monitor treatment efficacy. Combining knowledge from these transcriptome analyses with information from other omics approaches, such as metabolomics and single-cell sequencing, would provide comprehensive insight into these intricate host–microbe relationships and enable the development of personalized “microbiome-guided” treatments.

## 5. Conclusions

This study identified central host genes related to CRC-related signaling, genomic alterations, clinical characteristics and immune-cell infiltration. Some of these genes are suggested to be part of the host-response pathways associated with microbial dysbiosis by functional annotations. But the lack of microbiome data or experimental validation of molecular changes means that it is impossible to directly attribute the changes in molecules to microbes. These genes should therefore be regarded as hypotheses about host candidates that need to be tested in paired microbiome–host data sets and experimental systems. Future studies should determine the potential clinical relevance of these candidate genes using independent patient cohorts and appropriate experimental validation.

## Figures and Tables

**Table 1:** Top enriched biological pathways and processes identified by Metascape.

GO/Pathway ID	Category	Description	Count	%	Log10(*p*)	Log10(q)
R-HSA-1640170	Reactome Gene Sets	Cell cycle	217	8.98	−72.97	−68.63
R-HSA-1280215	Reactome Gene Sets	Cytokine signaling in immune system	197	8.15	−48.75	−44.89
R-HSA-2262752	Reactome Gene Sets	Cellular responses to stress	186	7.70	−41.65	−37.92
R-HSA-8953854	Reactome Gene Sets	Metabolism of RNA	179	7.41	−41.53	−37.89
GO:0006259	GO Biological Processes	DNA metabolic process	182	7.53	−40.34	−36.78
GO:0000278	GO Biological Processes	Mitotic cell cycle	149	6.17	−37.36	−33.98
GO:1901987	GO Biological Processes	Regulation of cell cycle phase transition	129	5.34	−33.53	−30.37
GO:0071396	GO Biological Processes	Cellular response to lipid	141	5.84	−32.22	−29.14
WP2004	WikiPathways	miR targeted genes in lymphocytes	115	4.76	−31.65	−28.59
GO:0019752	GO Biological Processes	Carboxylic acid metabolic process	168	6.95	−31.24	−28.20
R-HSA-68886	Reactome Gene Sets	M phase	111	4.59	−31.00	−28.00
GO:0051052	GO Biological Processes	Regulation of DNA metabolic process	124	5.13	−29.73	−26.78
WP2446	WikiPathways	Retinoblastoma gene in cancer	47	1.95	−27.56	−24.71
GO:0006979	GO Biological Processes	Response to oxidative stress	105	4.35	−27.35	−24.52
GO:0055086	GO Biological Processes	Nucleobase-containing small molecule metabolic process	133	5.50	−26.67	−23.85
GO:0008283	GO Biological Processes	Cell population proliferation	124	5.13	−25.05	−22.29
GO:0010638	GO Biological Processes	Positive regulation of organelle organization	116	4.80	−24.97	−22.22
GO:0001775	GO Biological Processes	Cell activation	150	6.21	−24.23	−21.53
GO:0045786	GO Biological Processes	Negative regulation of cell cycle	96	3.97	−24.15	−21.47
R-HSA-6798695	Reactome Gene Sets	Neutrophil degranulation	109	4.51	−23.93	−21.28

**Table 2:** Functional modules identified in the protein–protein interaction network.

GO	Description	Count	%	Log10(*p*)	Log10(q)
M39103	ZHONG PFC C1 MICROGLIA	101	4.20	−44.00	−40.00
M41703	FAN OVARY CL1 GPRC5A TNFRS12A HIGH SELECTABLE FOLLICLE STROMAL CELL	121	5.00	−41.00	−37.00
M41687	TRAVAGLINI LUNG PROLIFERATING NK T CELL	66	2.70	−37.00	−34.00
M41710	FAN OVARY CL8 MATURE CUMULUS GRANULOSA CELL 2	156	6.50	−36.00	−33.00
M39096	ZHONG PFC C1 OPC	88	3.60	−36.00	−32.00
M39036	FAN EMBRYONIC CTX NSC 2	87	3.60	−36.00	−32.00
M40010	BUSSLINGER GASTRIC ISTHMUS CELLS	126	5.20	−35.00	−32.00
M41717	FAN OVARY CL15 SMALL ANTRAL FOLLICLE GRANULOSA CELL	154	6.40	−35.00	−32.00
M40025	BUSSLINGER DUODENAL DIFFERENTIATING STEM CELLS	99	4.10	−34.00	−31.00
M39175	MURARO PANCREAS MESENCHYMAL STROMAL CELL	156	6.50	−33.00	−30.00
M39208	HAY BONE MARROW PRO B	96	4.00	−32.00	−29.00
M39041	FAN EMBRYONIC CTX MICROGLIA 1	66	2.70	−31.00	−28.00
M41711	FAN OVARY CL9 PUTATIVE APOPTOTIC ENDOTHELIAL CELL	104	4.30	−31.00	−28.00
M39059	MANNO MIDBRAIN NEUROTYPES HPROGBP	92	3.80	−30.00	−27.00
M39061	MANNO MIDBRAIN NEUROTYPES HPROGFPM	101	4.20	−30.00	−27.00
M39078	ZHONG PFC MAJOR TYPES NPCS	61	2.50	−29.00	−26.00
M41700	TRAVAGLINI LUNG OLR1 CLASSICAL MONOCYTE CELL	153	6.30	−28.00	−26.00
M41713	FAN OVARY CL11 MURAL GRANULOSA CELL	112	4.60	−28.00	−25.00
M39060	MANNO MIDBRAIN NEUROTYPES HPROGFPL	92	3.80	−27.00	−25.00
M41652	TRAVAGLINI LUNG PROXIMAL BASAL CELL	137	5.70	−27.00	−24.00

**Table 3:** Transcription factors potentially regulating candidate genes.

GO	Description	Count	%	Log10(*p*)	Log10(q)
M30019	HSD17B8 TARGET GENES	136	5.60	−24.00	−22.00
M102	E2F Q4 01	67	2.80	−20.00	−17.00
M8998	E2F1 Q4 01	65	2.70	−19.00	−17.00
M17117	E2F Q3 01	64	2.60	−18.00	−16.00
M9279	E2F1 Q6	62	2.60	−17.00	−15.00
M4220	E2F 03	64	2.60	−17.00	−15.00
M10498	TGCGCANK UNKNOWN	105	4.30	−16.00	−14.00
M10115	E2F Q4	61	2.50	−16.00	−14.00
M10526	E2F4DP1 01	61	2.50	−15.00	−13.00
M15729	E2F Q6 01	61	2.50	−15.00	−13.00
M12874	MYCMAX 01	63	2.60	−15.00	−13.00
M19064	E2F Q6	59	2.40	−15.00	−13.00
M3037	E2F1 Q6 01	60	2.50	−15.00	−13.00
M29900	BANP TARGET GENES	125	5.20	−15.00	−13.00
M12402	E2F1DP1 01	59	2.40	−15.00	−13.00
M12555	E2F1DP2 01	59	2.40	−15.00	−13.00
M17867	E2F 02	59	2.40	−15.00	−13.00
M19298	E2F4DP2 01	59	2.40	−15.00	−13.00
M17925	MGGAAGTG GABP B	126	5.20	−14.00	−12.00
M9645	GGGYGTGNY UNKNOWN	116	4.80	−14.00	−12.00

## Data Availability

The original contributions presented in this study are included in the article/Supplementary Material. Further inquiries can be directed to the corresponding author.

## Supplementary Materials

The following supporting information can be downloaded at: https://www.mdpi.com/article/10.3390/jcm15187131/s1, Table S1: Gene-to-annotation mapping of the Differentially Expressed Genes.

## References

[1] Ionescu V.A., Gheorghe G., Bacalbasa N., Chiotoroiu A.L., Diaconu C. (2023). Colorectal cancer: From risk factors to oncogenesis. Medicina.

[2] Klimeck L., Heisser T., Hoffmeister M., Brenner H. (2023). Colorectal cancer: A health and economic problem. Best Pract. Res. Clin. Gastroenterol..

[3] Shah S.C., Itzkowitz S.H. (2022). Colorectal cancer in inflammatory bowel disease: Mechanisms and management. Gastroenterology.

[4] Mármol I., Sánchez-de-Diego C., Pradilla Dieste A., Cerrada E., Rodriguez Yoldi M.J. (2017). Colorectal carcinoma: A general overview and future perspectives in colorectal cancer. Int. J. Mol. Sci..

[5] Schmitt M., Greten F.R. (2021). The inflammatory pathogenesis of colorectal cancer. Nat. Rev. Immunol..

[6] Qu R., Zhang Y., Ma Y., Zhou X., Sun L., Jiang C., Zhang Z., Fu W. (2023). Role of the gut microbiota and its metabolites in tumorigenesis or development of colorectal cancer. Adv. Sci..

[7] Canellas-Socias A., Sancho E., Batlle E. (2024). Mechanisms of metastatic colorectal cancer. Nat. Rev. Gastroenterol. Hepatol..

[8] Shin A.E., Giancotti F.G., Rustgi A.K. (2023). Metastatic colorectal cancer: Mechanisms and emerging therapeutics. Trends Pharmacol. Sci..

[9] Roshandel G., Ghasemi-Kebria F., Malekzadeh R. (2024). Colorectal cancer: Epidemiology, risk factors, and prevention. Cancers.

[10] Shakhpazyan N.K., Mikhaleva L.M., Bedzhanyan A.L., Gioeva Z.V., Mikhalev A.I., Midiber K.Y., Pechnikova V.V., Biryukov A.E. (2024). Exploring the role of the gut microbiota in modulating colorectal cancer immunity. Cells.

[11] Nunes L., Li F., Wu M., Luo T., Hammarström K., Torell E., Ljuslinder I., Mezheyeuski A., Edqvist P.H., Löfgren-Burström A. (2024). Prognostic genome and transcriptome signatures in colorectal cancers. Nature.

[12] Wang F., Long J., Li L., Wu Z.X., Da T.T., Wang X.Q., Huang C., Jiang Y.H., Yao X.Q., Ma H.Q. (2023). Single-cell and spatial transcriptome analysis reveals the cellular heterogeneity of liver metastatic colorectal cancer. Sci. Adv..

[13] Zhang Q., Liu Y., Wang X., Zhang C., Hou M., Liu Y. (2023). Integration of single-cell RNA sequencing and bulk RNA transcriptome sequencing reveals a heterogeneous immune landscape and pivotal cell subpopulations associated with colorectal cancer prognosis. Front. Immunol..

[14] Clough E., Barrett T., Wilhite S.E., Ledoux P., Evangelista C., Kim I.F., Tomashevsky M., Marshall K.A., Phillippy K.H., Sherman P.M. (2024). NCBI GEO: Archive for gene expression and epigenomics data sets: 23-year update. Nucleic Acids Res..

[15] Barrett T., Wilhite S.E., Ledoux P., Evangelista C., Kim I.F., Tomashevsky M., Marshall K.A., Phillippy K.H., Sherman P.M., Holko M. (2012). NCBI GEO: Archive for functional genomics data sets—Update. Nucleic Acids Res..

[16] Chung P.J., Bohme J.F., Mecklenbrauker C.F., Hero A.O. (2007). Detection of the number of signals using the Benjamini-Hochberg procedure. IEEE Trans. Signal Process..

[17] Huang D.W., Sherman B.T., Lempicki R.A. (2009). Systematic and integrative analysis of large gene lists using DAVID bioinformatics resources. Nat. Protoc..

[18] Zhou Y., Zhou B., Pache L., Chang M., Khodabakhshi A.H., Tanaseichuk O., Benner C., Chanda S.K. (2019). Metascape provides a biologist-oriented resource for the analysis of systems-level datasets. Nat. Commun..

[19] Szklarczyk D., Nastou K., Koutrouli M., Kirsch R., Mehryary F., Hachilif R., Hu D., Peluso M.E., Huang Q., Fang T. (2025). The STRING database in 2025: Protein networks with directionality of regulation. Nucleic Acids Res..

[20] Shannon P., Markiel A., Ozier O., Baliga N.S., Wang J.T., Ramage D., Amin N., Schwikowski B., Ideker T. (2003). Cytoscape: A software environment for integrated models of biomolecular interaction networks. Genome Res..

[21] Smoot M.E., Ono K., Ruscheinski J., Wang P.L., Ideker T. (2011). Cytoscape 2.8: New features for data integration and network visualization. Bioinformatics.

[22] Chin C.-H., Chen S.H., Wu H.H., Ho C.W., Ko M.T., Lin C.Y. (2014). cytoHubba: Identifying hub objects and sub-networks from complex interactome. BMC Syst. Biol..

[23] Tang Z., Kang B., Li C., Chen T., Zhang Z. (2019). GEPIA2: An enhanced web server for large-scale expression profiling and interactive analysis. Nucleic Acids Res..

[24] Li T., Fan J., Wang B., Traugh N., Chen Q., Liu J.S., Li B., Liu X.S. (2017). TIMER: A web server for comprehensive analysis of tumor-infiltrating immune cells. Cancer Res..

[25] Gao J., Aksoy B.A., Dogrusoz U., Dresdner G., Gross B., Sumer S.O., Sun Y., Jacobsen A., Sinha R., Larsson E. (2013). Integrative analysis of complex cancer genomics and clinical profiles using the cBioPortal. Sci. Signal..

[26] Liu C.-J., Hu F.F., Xie G.Y., Miao Y.R., Li X.W., Zeng Y., Guo A.Y. (2023). GSCA: An integrated platform for gene set cancer analysis at genomic, pharmacogenomic and immunogenomic levels. Brief. Bioinform..

[27] Wang C., Sandhu J., Tsao A., Fakih M. (2022). Presence of concurrent TP53 mutations is necessary to predict poor outcomes within the SMAD4 mutated subgroup of metastatic colorectal cancer. Cancers.

[28] Yan H., Jiang F., Yang J. (2022). Association of β-Catenin, APC, SMAD3/4, Tp53, and Cyclin D1 Genes in Colorectal Cancer: A Systematic Review and Meta-Analysis. Genet. Res..

[29] Amani M.S., Peymani M. (2024). Investigating the impact of SMAD2 and SMAD4 downregulation in colorectal cancer and their correlation with immune markers, prognosis, and drug resistance and sensitivity. Mol. Biol. Rep..

[30] Lahoz S., Rodríguez A., Fernandez L., Gorría T., Moreno R., Esposito F., Oliveres H., Albiol S., Sauri T., Pesantez D. (2022). Mutational status of SMAD4 and FBXW7 affects clinical outcome in TP53–mutated metastatic colorectal cancer. Cancers.

[31] Kumar R., Mahmoud M.M., Tashkandi H.M., Haque S., Harakeh S., Ponnusamy K., Haider S. (2023). Combinatorial network of transcriptional and miRNA regulation in colorectal cancer. Int. J. Mol. Sci..

[32] Tan C.H., Lim S.H., Sim K.S. (2026). Computational Elucidation of hub genes and pathways correlated with the development of 5-Fluorouracil resistance in HCT 116 colorectal carcinoma cell line. Biochem. Genet..

[33] Cheng B., Xu L., Zhang Y., Yang H., Liu S., Ding S., Zhao H., Sui Y., Wang C., Quan L. (2024). Correlation between NGS panel-based mutation results and clinical information in colorectal cancer patients. Heliyon.

[34] Michas A., Michas V., Anagnostou E., Galanopoulos M., Tolia M., Tsoukalas N. (2023). The clinical significance of microRNAs in colorectal cancer signaling pathways: A review. Glob. Med. Genet..

[35] Tahara T., Yamamoto E., Suzuki H., Maruyama R., Chung W., Garriga J., Jelinek J., Yamano H.O., Sugai T., An B. (2014). Fusobacterium in colonic flora and molecular features of colorectal carcinoma. Cancer Res..

[36] Zou S., Yang C., Zhang J., Zhong D., Meng M., Zhang L., Chen H., Fang L. (2024). Multi-omic profiling reveals associations between the gut microbiome, host genome and transcriptome in patients with colorectal cancer. J. Transl. Med..

[37] Diaz F.C., Waldrup B., Carranza F.G., Manjarrez S., Velazquez-Villarreal E. (2025). Artificial intelligence-enhanced precision medicine reveals prognostic impact of TGF-beta pathway alterations in FOLFOX-treated early-onset colorectal cancer among disproportionately affected populations. Int. J. Mol. Sci..

[38] Stoia O., Anderco P., Badiu T., Todor S.B., Ichim C. (2026). Artificial intelligence in optimizing antimicrobial therapy for gastro-renal disorders. Front. Cell. Infect. Microbiol..

[39] Mohammadpour S., Torshizi Esfahani A., Sarpash S., Vakili F., Zafarjafarzadeh N., Mashaollahi A., Pardakhtchi A., Nazemalhosseini-Mojarad E. (2024). Hippo signaling pathway in colorectal cancer: Modulation by various signals and therapeutic potential. Anal. Cell. Pathol..

[40] Martinez-Canales R., Montoya-Rosales A., Molé A., Salinas-Carmona M.C., Macias-Segura N. (2025). ABS0257 IDENTIFICATION OF KEY OVEREXPRESSED HUB GENES IN DERMATOMYOSITIS-ASSOCIATED CANCERS AS POTENTIAL SCREENING BIOMARKERS. Ann. Rheum. Dis..

[41] Bichalski B., Bichalska-Lach M., Waniczek D. (2025). Diagnostic Pathways and Molecular Biomarkers in Colorectal Cancer: Current Evidence and Perspectives in Poland. Curr. Issues Mol. Biol..

[42] Atasever S. (2024). Identification of potential hub genes as biomarkers for breast, ovarian, and endometrial cancers. Front. Life Sci. Relat. Technol..

[43] Taieb J., Svrcek M., Cohen R., Basile D., Tougeron D., Phelip J.M. (2022). Deficient mismatch repair/microsatellite unstable colorectal cancer: Diagnosis, prognosis and treatment. Eur. J. Cancer.

[44] Li J., Zhu N., Wang C., You L., Guo W., Yuan Z., Qi S., Zhao H., Yu J., Huang Y. (2023). Preoperative albumin-to-globulin ratio and prognostic nutritional index predict the prognosis of colorectal cancer: A retrospective study. Sci. Rep..

